# *Schisandra* Lignan Extract Protects against Carbon Tetrachloride-Induced Liver Injury in Mice by Inhibiting Oxidative Stress and Regulating the NF-*κ*B and JNK Signaling Pathways

**DOI:** 10.1155/2017/5140297

**Published:** 2017-01-26

**Authors:** Qingshan Chen, Qi Zhan, Ying Li, Sen Sun, Liang Zhao, Hai Zhang, Guoqing Zhang

**Affiliations:** Department of Pharmacy, Shanghai Eastern Hepatobiliary Surgery Hospital, Second Military Medical University, Shanghai 200438, China

## Abstract

*Schisandra chinensis* (*S. chinensis*) is a traditional Chinese herbal medicine widely used for the treatment of liver disease, whose main active components are lignans. However, the action mechanisms of the lignans in* S. chinensis* remain unclear. This study aimed to investigate the protective effect and related molecular mechanism of* Schisandra* lignan extract (SLE) against carbon tetrachloride- (CCl_4_-) induced acute liver injury in mice. Different doses of SLE at 50, 100, and 200 mg/kg were administered daily by gavage for 5 days before CCl_4_ treatment. The results showed that SLE significantly decreased the activities of serum ALT/AST and reduced liver pathologic changes induced by CCl_4_. Pretreatment with SLE not only decreased the content of MDA but increased SOD, GSH, and GSH-Px activities in the liver, suggesting that SLE attenuated CCl_4_-induced oxidative stress. The expression levels of inflammatory cytokines TNF-a, IL-1*β*, and IL-6 were decreased after oral administration of SLE, probably because lignans inhibited the NF-*κ*B activity. Additionally, SLE also inhibited hepatocyte apoptosis by suppressing JNK activation and regulating Bcl-2/Bax signaling pathways. In conclusion, these results suggested that SLE prevented CCl_4_-induced liver injury through a combination of antioxidative stress, anti-inflammation, and antihepatocyte apoptosis and alleviated inflammation and apoptosis by regulating the NF-*κ*B, JNK, and Bcl-2/Bax signaling pathways.

## 1. Introduction

Liver injury has been recognized as a serious health problem worldwide, which can be caused by viral infections, hepatotoxic drugs, and toxic chemicals [1, 2]. There are few effective drugs available for the clinical treatment of acute and chronic liver injury at present. It is necessary to elucidate the possible molecular mechanism underlying liver injury for the sake of developing effective drugs [3]. Carbon tetrachloride (CCl_4_) is a hepatotoxic chemical that has been widely used to induce experimental liver injury models [4–6]. Metabolic activation of CCl_4_ to reactive free radicals by cytochrome P450 enzymes is the initiating event of CCl_4_-induced liver injury. These free radicals result in lipid peroxidation of hepatocellular membrane, ultimately leading to acute liver inflammation and hepatocyte death [4, 7].

It is widely believed that liver injury is closely associated with oxidative stress, inflammation, and apoptosis in the process of CCl_4_-induced liver damage [7–9]. Several studies have demonstrated that persistent inflammatory response contributes to the progression of chronic liver diseases [10]. Nuclear factor kappa B (NF-*κ*B) is a key transcription factor that plays an important role in regulating cell growth, differentiation, apoptosis, and inflammation. Under pathological conditions, NF-*κ*B is activated to promote inflammatory response by inducing the expression of a series of proinflammatory cytokines, such as tumor necrosis factor *α* (TNF-a), interleukin-1*β* (IL-1*β*), and IL-6 [11]. Thus, downregulation of NF-*κ*B activity may serve to inhibit the production of proinflammatory cytokines and attenuate inflammatory response. Additionally, previous studies have confirmed that oxidative stress-mediated ROS can induce apoptosis by activating c-Jun N-terminal kinase (JNK) signaling pathway [12]. It was reported that JNK can promote apoptosis by directly activating mitochondrial apoptotic proteins [13]. In recent studies, JNK activation was reported to play a critical role in the CCl_4_-induced liver injury [14, 15], suggesting that JNK may be a potential target of liver injury. Theoretically, any compound that attenuates oxidative stress, inflammation, and apoptosis in the liver can protect or eliminate liver injury.

*Schisandra chinensis* (*S. chinensis*) is a traditional Chinese herbal medicine that has been used for the treatment of viral and chemical liver injury in Asia for thousands of years [16–18]. The lignans as the main active ingredients in* S. chinensis* have various pharmacological effects such as antioxidative, anti-inflammatory, antitumor, and hepatoprotective activities [18, 19]. It was reported that* S. chinensis* exerted its effect against chemical-induced liver injury by regulating the antioxidation function and heat shock proteins [20, 21]. However, further mechanisms underlying the protective effect of* S. chinensis* against CCl_4_-induced liver injury remain unclear, and whether this protective effect is associated with inhibition of inflammation and apoptosis needs to be further clarified. The present study aimed to evaluate the effect of* Schisandra* lignan extract (SLE) on oxidative stress, inflammation, and hepatocyte apoptosis after CCl_4_ treatment and investigate inflammation and hepatocyte apoptosis related signaling pathways to explore its possible molecular mechanism.

## 2. Materials and Methods

### 2.1. Chemicals and Reagents

In this experiment, CCl_4_ was purchased from Jiangsu Qiangsheng Chemical (Jiangsu, China). The positive drug Bicyclol was obtained from Beijing union pharmaceutical factory (Beijing, China). The detection kits for superoxide dismutase (SOD), glutathione peroxidase (GSH-Px), glutathione (GSH), and malondialdehyde (MDA) were all purchased from Nanjing Jiancheng Institute of Biotechnology (Nanjing, China). Enzyme-linked immunosorbent assay kits for mouse TNF-a were purchased from R&D Systems (Minneapolis, USA). Monoclonal anti-NF-*κ*B rabbit antibody was purchased from Santa Cruz Biotechnology Inc. (Santa Cruz, CA). Monoclonal anti-Bcl-2, anti-Bax, anti-cleaved caspase-3, and GAPDH antibodies were obtained from Cell Signaling Technology (Danvers, MA). In addition, antibodies against JNK, p-JNK, and p-NF-*κ*B were also from Cell Signaling Technology (Danvers, MA). CYP2E1 antibodies were purchased from Sangon Biotechnology (Shanghai, China). Secondary antibodies used in Western blot were IRDye® 800CW anti-rabbit IgG (H+L) (LI-COR Biosciences) and IRDye 800CW anti-mouse IgG (H+L) (LI-COR Biosciences).

### 2.2. Preparation of Schisandra Lignan Extract

Firstly, the air-dried* S. chinensis* raw material was powdered and extracted twice with ethanol (95%) for 2 h under reflux at 50°C. The filtered sample was extracted 3 times with ethyl acetate successively. The extracts were concentrated under reduced pressure at 37°C in a rotator evaporator. Then, the mixture was subjected to normal phase column chromatography and eluted with petroleum ether and ethyl acetate. The extracts were collected and evaporated under reduced pressure. Finally, the SLE was produced and maintained at 4°C for further study.

### 2.3. Animals and Experimental Design

Male C57BL/6 mice weighing  20 ± 2 g (Shanghai SLAC Laboratory Animal Center, Shanghai, China) were housed under appropriate conditions (25 ± 2°C and 12-hour light/dark cycle) with free access to water and food. All animal procedures were performed in accordance with the Guide for the Care and Use of Laboratory Animals and approved by the Animal Experimental Ethics Committee of the Second Military Medical University (Shanghai, China).

After one-week environmental adaptation, 60 mice were equally randomized into six groups: (1) control group, (2) model (CCl_4_) group, (3) SLE (50 mg/kg) + CCl_4_ group, (4) SLE (100 mg/kg) + CCl_4_ group, (5) SLE (200 mg/kg) + CCl_4_ group, and (6) Bicyclol (200 mg/kg) + CCl_4_ group. SLE solution was prepared by suspending it in 0.5% (w/v) sodium carboxymethyl cellulose (CMC-Na). Mice were fed SLE solution (50, 100, and 200 mg/kg) daily for 5 days by intragastric administration; mice in the control and model groups were given 0.5% CMC-Na solution. Two hours after the last dose of SLE, mice in the model group and SLE + CCl_4_ group were injected intraperitoneally with 0.3% CCl_4_ (15 mL/kg, in olive oil). Subsequently, all mice were fasted overnight and sacrificed 24 h after CCl_4_ injection. Serum samples and liver tissues were harvested, a portion of the liver tissue was immediately fixed in 10% formalin for pathological examination, and the remaining liver was stored at −80°C for further analysis.

### 2.4. ALT/AST Assessment

Serum samples were separated from blood by centrifugation at 3000 ×g for 15 min at 4°C. Serum alanine transaminase (ALT) and aspartate transaminase (AST) were determined by using a clinical automatic analyzer (Hitachi, Japan) and commercial reagent kit (Roche Diagnostic, Mannheim, Germany) according to the manufacturer's protocol.

### 2.5. Determination of MDA, SOD, GSH, and GSH-Px Activity

The liver tissue was homogenized in 9 volumes of 0.9% saline solution and centrifuged at 3000 ×g for 15 min at 4°C. The supernatant was used for determination of MDA, SOD, GSH-Px, and GSH according to the protocols of commercially available kits.

### 2.6. H&E Staining and TUNEL Assay

The liver tissue was fixed in 10% buffered formalin for 2 days, paraffin-embedded, sliced cut into 5 *μ*m thick sections, and stained with haematoxylin/eosin (H&E) according to a standard protocol. The liver sections were imaged under a microscope (Olympus, Japan). TUNEL staining was performed according to the manual protocols (Boster, MK1020). Nuclei were stained with DAPI for the assessment of nuclear morphology. All slices were imaged with a fluorescence microscope (magnification, 400x). Three slices in each group were randomly selected to count the positive cells.

### 2.7. Real-Time PCR Analysis

Total RNA was extracted from the liver tissue using the TRIzol reagent (Invitrogen, 15596026) according to the standard protocol. The first-strand cDNA was synthesized by PrimeScript RT reagent kit (Takara, #6210A), and total RNA (2 *μ*g) was using as a template. The target mRNA expression was quantified with SYBER Green PCR Master Mix (Takara, #RR420Q) using Step One Real-Time PCR System (Applied Biosystems, Warrington, UK). The conditions of real-time PCR analysis were as follows: (1) holding stage: 95°C, 30 s; (2) cycling Stage: 95°C, 5 s; 60°C, 34 s; 40 cycles; (3) melt curve stage: 95°C, 15 s; 60°C, 1 min; 95°C, 15 s. GAPDH was amplified as reference genes. The primer sequences used in PCR are shown in Table 1. The expression levels were measured in terms of the cycle threshold (Ct) and then normalized to GAPDH expression using the 2^−ΔΔCt^ method [22].

### 2.8. Western Blotting Analysis

Total proteins were extracted from the liver tissue using the strong RIPA lysate kit (Beyotime Biotechnology, P0013B) containing 1 mM PMSF (Beyotime Biotechnology, ST506), and the protein concentration was quantified with the BCA protein kit (Thermo Scientific, 23228). Then, Western blotting assays were performed as previously described. An equal amount of protein (60 *μ*g) was denatured by mixing with corresponding loading buffer at 100°C for 5 min. An equivalent volume of the supernatant was loaded onto SDS-polyacrylamide gels (10%) for electrophoretic separation and then transferred onto nitrocellulose membranes (Millipore, HATF00010). After being blocked with 5% skim milk (BD, 232100) for 2 h, the membranes were incubated with primary antibodies at 4°C overnight and then with the secondary antibodies for 2 h at room temperature. Protein expression was imaged by Odyssey Infrared Imaging System (LI-COR Biosciences, USA).

### 2.9. Statistical Analysis

Data are presented as the mean ± SEM. Differences between groups were determined by a two-tailed Student's *t*-test in GraphPad Prism 5. *P* < 0.05 was considered statistically significant.

## 3. Results

### 3.1. Determination of Components in SLE by HPLC-UV

The SLE content was detected by Agilent 1100 series HPLC system (Agilent Technologies, USA). Chromatographic separation was performed by using an Agilent Zorbax SB-C_18_ column (3.0 × 100 mm, 3.5 *μ*m), and a gradient elution of solvent A (water) and solvent B (acetonitrile) was performed as follows: 0–10 min, 40–58% B; 10–15 min, 58–60% B; 15–20 min, 60–70% B; 20–25 min, 70–95% B; 25–30 min, 95% B. The flowing rate was 0.4 mL/min and column temperature was maintained at 30°C. The detector wavelength was set at 225 nm and the injection volume was 5 *μ*L.

**Figure 1:**
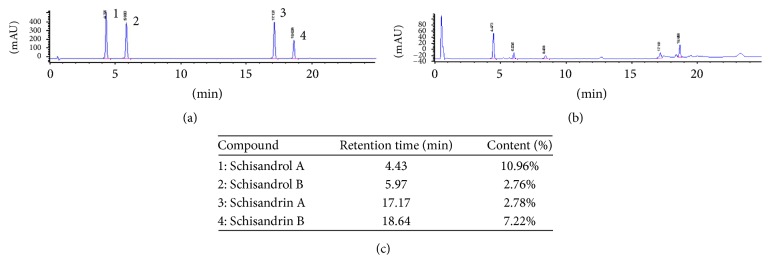
Determination of four components in* Schisandra* lignan extract (SLE) by HPLC-UV. (a) HPLC-UV chromatogram of four lignan standards. (b) HPLC-UV chromatogram of SLE sample. (c) The content of lignans in SLE. 1: schisandrol A; 2: schisandrol B; 3: schisandrin A; 4: schisandrin B.

As shown in Figure 1, the total lignan content of SLE was over 50% (W/W), in which schisandrol A was 10.96%, schisandrol B was 2.76%, schisandrin A was 2.78%, and schisandrin B 7.22%.

### 3.2. SLE Protects against CCl_4_-Induced Acute Liver Injury

**Figure 2:**
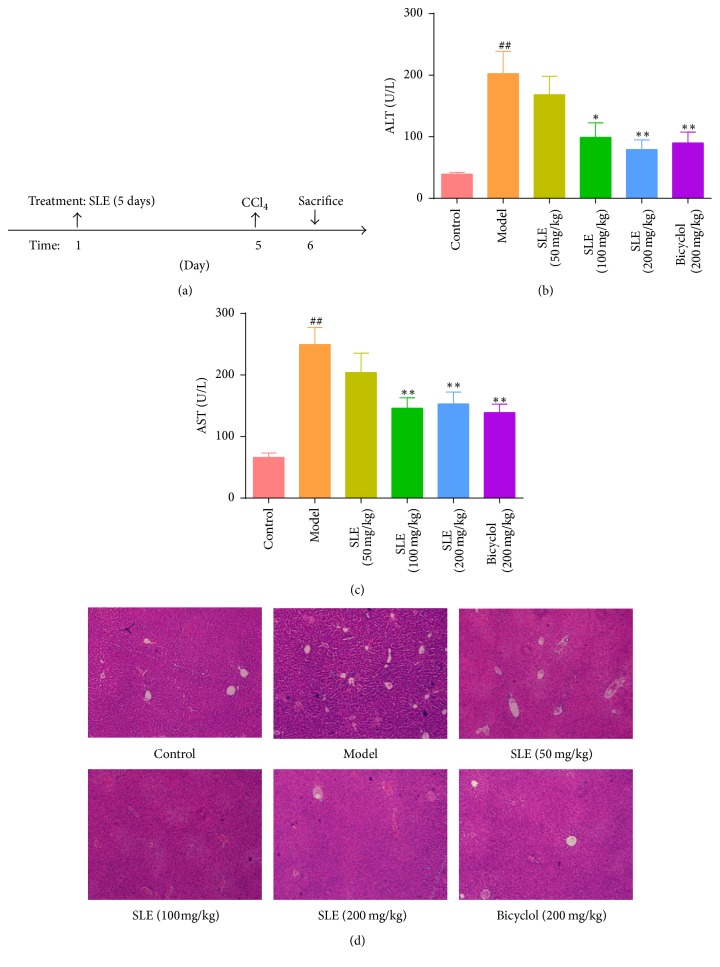
SLE protects against CCl_4_-induced acute liver injury. (a) The experimental design scheme; (b, c) serum ALT and AST activities; (d) H&E staining of liver sections (original magnification, 40x). Data are expressed as the mean ± SEM (*n* = 8). ^##^*P* < 0.01  versus control group. ^*∗*^*P* < 0.05  and ^*∗∗*^*P* < 0.01  versus model group.

Liver injury was evaluated by serum ALT/AST activities and histopathological analysis. As expected, severe liver injury was induced by CCl_4_ treatment in the mice, as indicated by elevation of ALT/AST activities and histopathological analysis of H&E staining. The levels of ALT and AST were 39.40 ± 7.76 U/L and 66.20 ± 21.54 U/L, respectively, in the control group versus 202.70 ± 113.50 U/L and 249.60 ± 87.01 U/L in the model group (Figures 2(b) and 2(c)). In agreement with serum ALT/AST activities, liver damage was observed in the liver sections as evidenced by inflammatory cell infiltration, hepatocyte ballooning degeneration, and necrosis after CCl_4_ treatment. However, pretreatment with SLE for 5 days not only decreased the upregulation of ALT/AST levels but ameliorated pathological lesions induced by CCl_4_ (Figure 2(d)). Taken together, these data suggest that SLE has a protective effect against CCl_4_-induced acute liver injury in mice.

### 3.3. SLE Attenuates CCl_4_-Induced Oxidative Stress

**Figure 3:**
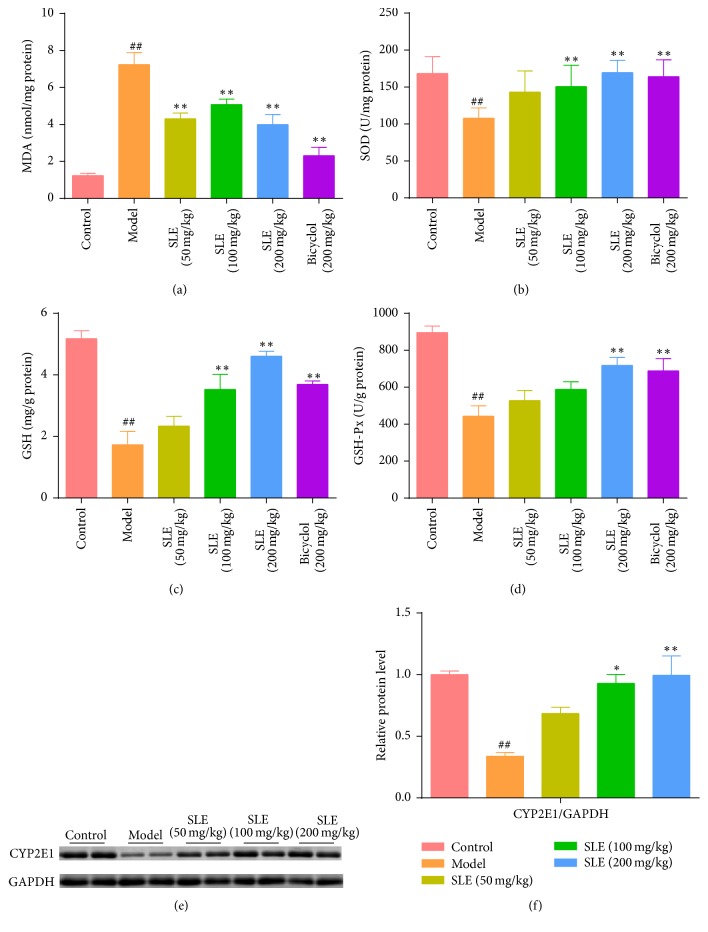
SLE attenuates CCl_4_-induced oxidative stress. (a) Lipid peroxidation MDA; (b) antioxidant enzyme SOD; (c) liver GSH levels; (d) liver GSH-Px activities. (e) Western blot analysis of CYP2E1 levels in the liver. (f) Densitometric analysis of Western blots. Data are expressed as the mean ± SEM (*n* = 8). ^##^*P* < 0.01 versus control group. ^*∗*^*P* < 0.05 and ^*∗∗*^*P* < 0.01 versus model group.

In this study, the activities of MDA, SOD, GSH, and GSH-Px were detected to investigate the effects of SLE on liver oxidative stress caused by CCl_4_. Malondialdehyde (MDA) is the product of cell membrane lipid peroxidation and often used as a biomarker of liver oxidative damage. After CCl_4_ treatment, the content of MDA increased from  1.24 ± 0.27 nmol/mg protein to  7.25 ± 1.82 nmol/mg protein in the liver, indicating that oxidative liver injury was induced by CCl_4_ in the mice. Interestingly, SLE significantly decreased the MDA content 2-fold in response to 200 mg/kg SLE treatment (Figure 3(a)). Compared with the control group, the activity of SOD was decreased (*P* < 0.05) in the model group, which was obviously reversed by 100 or 200 mg/kg SLE (Figure 3(b)). Additionally, the activities of GSH and GSH-Px declined after CCl_4_ treatment in the model group (1.73 ± 1.24 mg/g protein; 443.50 ± 158.50 U/mg protein) compared to control (5.18 ± 0.57 mg/g protein; 896.20 ± 77.54 U/mg protein). Conversely, SLE pretreatment markedly increased GSH and GSH-Px activities in a dose-dependent manner compared with model group (Figures 3(c) and 3(d)). We Know that CCl_4_ are primarily metabolized and activated by cytochrome P450 2E1 (CYP2E1) and thus SLE could attenuate CCl_4_-induced oxidative stress by inhibiting the expression of CYP2E1. As expected, the expression of CYP2E1 was decreased after CCl_4_ treatment, but treatment with SLE significantly increased its expression (Figures 3(e) and 3(f)). These results indicate that SLE could suppress CCl_4_-induced oxidative stress maybe through regulating CYP2E1 activity.

### 3.4. SLE Suppresses CCl_4_-Induced Inflammatory Response and NF-*κ*B Activation

**Figure 4:**
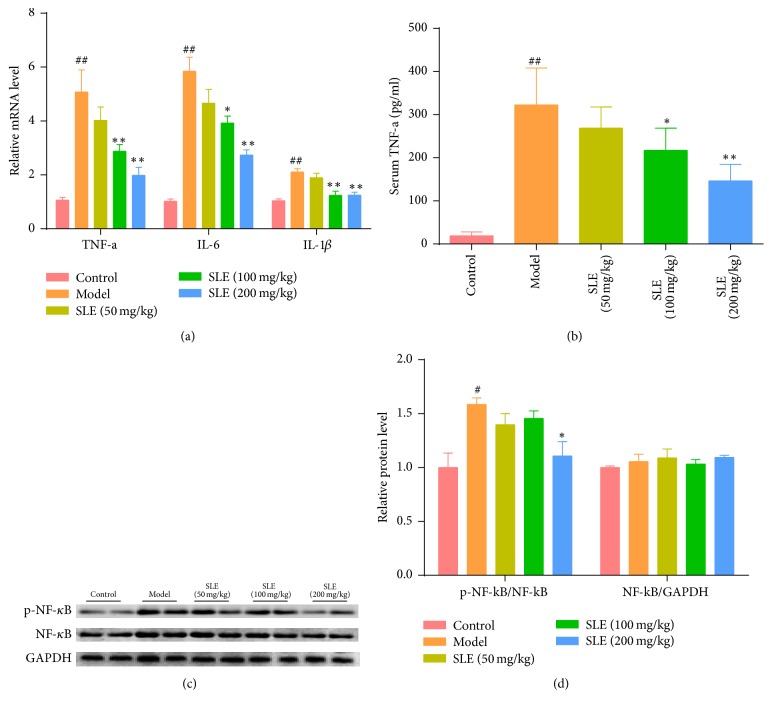
SLE suppresses CCl_4_-induced inflammatory response by inhibiting NF-*κ*B activation. (a) Relative mRNA level of TNF-a, IL-6, and IL-1*β* (*n* = 6); (b) serum TNF-a levels (*n* = 6). (c) Western blot analysis was performed to measure p-NF-*κ*B and NF-*κ*B. (d) Densitometric analysis of Western blots. ^#^*P* < 0.05 and ^##^*P* < 0.01 versus control group. ^*∗*^*P* < 0.05 and ^*∗∗*^*P* < 0.01 versus model group.

Knowing that liver injury is associated with inflammatory response, the effect of SLE on the expression of inflammatory cytokines induced by CCl_4_ was evaluated. Compared with the control group, the expressions of hepatic TNF-a, IL-1*β*, and IL-6 mRNA were significantly elevated after CCl_4_ treatment (*P* < 0.01), whereas these inflammatory cytokines were reduced after 100 or 200 mg/kg SLE treatment (Figure 4(a)). Moreover, the level of serum TNF-a increased more than 10-fold after CCl_4_ injection in the model group, but SLE treatment blocked this trend especially at the dose of 200 mg/kg in TNF-a level (Figure 4(b)).

Since the NF-*κ*B signaling pathway plays an important role in inflammation, we investigated whether NF-*κ*B signaling was inhibited when mice were treated with SLE. The Western blot results showed that CCl_4_ treatment increased the p-NF-*κ*B expression in the liver compared with control mice. However, SLE treatment inhibited p-NF-*κ*B activity in CCl_4_-treated mice. All these results indicate that SLE could attenuate the release of proinflammatory cytokines and inhibit the activation of NF-*κ*B in the CCl_4_-induced liver injury (Figures 4(c) and 4(d)).

### 3.5. SLE Decreases CCl_4_-Induced Hepatocytes Apoptosis

**Figure 5:**
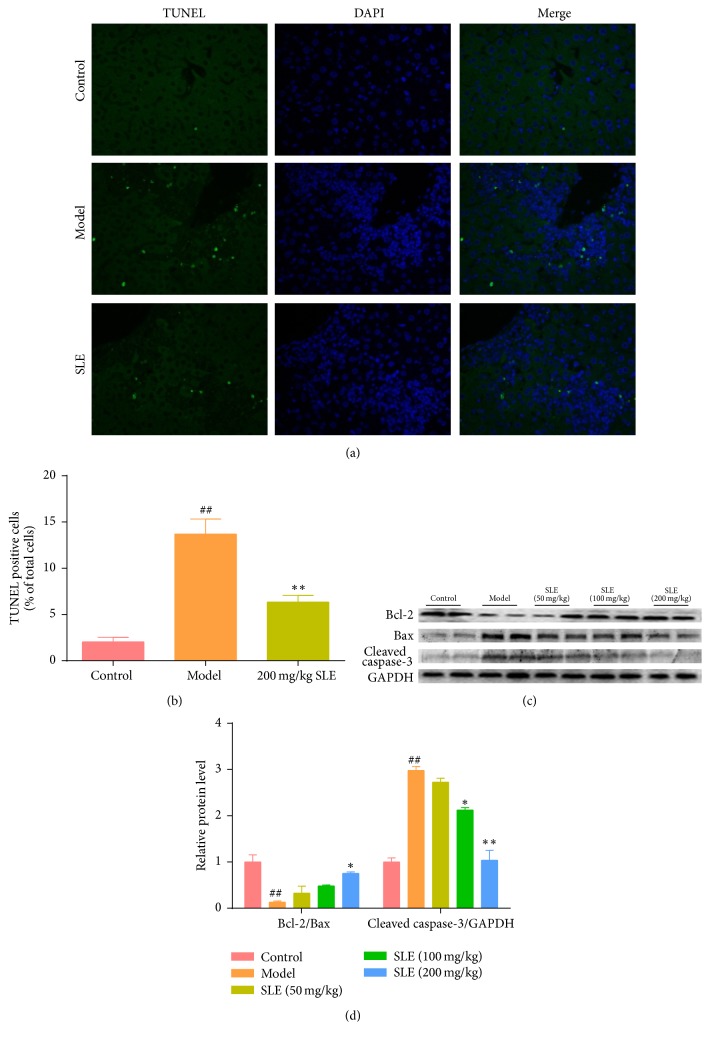
SLE decreases CCl_4_-induced hepatocytes apoptosis. (a) TUNEL stained liver sections (magnification, 400x); green fluorescence indicated the positive cells. (b) Statistic analysis of the relative proportion of TUNEL-positive cells in the liver (*n* = 3). (c) Western blot analysis of Bax, Bcl-2, and cleaved caspase-3 levels in the liver. (d) Densitometric analysis of Western blots. ^##^*P* < 0.01 versus control group. ^*∗*^*P* < 0.05 and ^*∗∗*^*P* < 0.01 versus model group.

To validate the possible role of SLE on CCl_4_-induced apoptosis, TUNEL assay was performed to investigate cell apoptosis. The number of TUNEL-positive cells in the liver of CCl_4_-treated mice was significantly increased more than 8-fold compared with normal mice. However, the number of liver TUNEL-positive cells was decreased after 200 mg/kg SLE treatment as compared with the model group (*P* < 0.01) (Figures 5(a) and 5(b)). To further identify the effect of SLE on apoptosis, the expression of apoptotic protein cleaved caspase-3 was determined by Western bolting. As shown in Figure 5(c), the expression level of cleaved caspase-3 was significantly elevated in the CCl_4_-treated mice. However, SLE treatment inhibited this elevation in a dose-dependent manner. Next, in order to determine whether the antiapoptotic effect of SLE was associated with mitochondrial apoptosis, Bcl-2 and Bax were determined. The results showed that the expression level of Bcl-2 was decreased, whereas Bax was apparently increased in the model group, leading to Bcl-2/Bax ratio imbalance. Interestingly, SLE treatment decreased the expression level of Bax and increased the expression level of Bcl-2, thus recovering the Bcl-2/Bax ratio balance (Figures 5(c) and 5(d)).

### 3.6. SLE Suppresses JNK Signaling Pathway in CCl_4_-Induced Liver Injury

**Figure 6:**
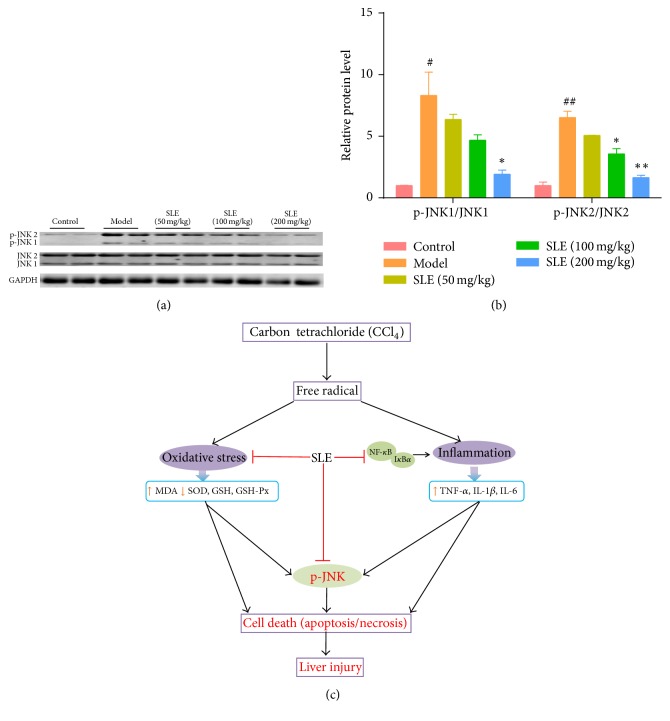
SLE suppresses JNK signaling pathway. (a) Western blot analysis of total JNK and phosphor-JNK levels in the liver. (b) Densitometric analysis of Western blots. (c) A diagram shows the possible mechanism of the SLE against CCl_4_-induced liver injury in mice. ^#^*P* < 0.05 and ^##^*P* < 0.01 versus control group. ^*∗*^*P* < 0.05 and ^*∗∗*^*P* < 0.01 versus model group.

Knowing that JNK activation is a critical event of mitochondrial dysfunction involved in CCl_4_-induced hepatotoxicity, the expression level of JNK and p-JNK was detected to determine whether SLE could inhibit JNK activation against hepatocyte death. The expression of total JNK in the liver was similar between each group, while the expression of phosphorylated JNK significantly increased in the model group as compared with the control group. In addition, compared with JNK1, JNK2 appeared to be markedly activated after CCl_4_ challenge. Moreover, SLE treatment dramatically reduced CCl_4_-induced increase in the expression of p-JNK protein especially at the dose of 200 mg/kg (Figures 6(a) and 6(b)).

## 4. Discussion

*S. chinensis* is a traditional Chinese herbal medicine that has been widely used as a tonic and medicinal ingredient in China. In this study, we investigate the protective effect and possible molecular mechanism of SLE against CCl_4_-induced acute liver injury in mice. The results of this study showed that SLE could attenuate liver injury via inhibiting oxidative stress, inflammation, and apoptosis process in mice (summarized in Figure 6(c)). In the model group, the activities of ALT/AST were markedly increased after CCl_4_ treatment. Histopathological analysis of H&E staining also reflected the severity of CCl_4_-induced liver injury. When SLE was administered before CCl_4_ injection, mice were protected against CCl_4_ hepatotoxicity as evidenced by lower serum ALT/AST levels and improved liver morphology and histology. These results confirm the hepatoprotective effect of SLE as reported previously.

Oxidative stress is the crucial step in the development of CCl_4_-induced liver injury [23]. CCl_4_ is known to be metabolized to trichloromethyl free radicals (•CCl_3_) by cytochrome P450 enzymes [24, 25] and these free radicals may attack intracellular nucleic acid, protein, and lipid, ultimately leading to hepatocyte oxidative damage and death [4]. MDA is the end product of lipid peroxidation and often used as a biomarker of liver oxidative damage. It was found in this study that SLE pretreatment significantly decreased the level of liver MDA induced by CCl_4_ in mice, indicating that SLE could prevent CCl_4_-induced lipid peroxidation. SOD is an important antioxidant enzyme by transforming superoxide radical to H_2_O_2_, while GSH and GSH-Px could degrade H_2_O_2_ to protect cells against oxidative injury. Previous reports showed that* S. chinensis* extract and its lignans stimulated GSH related enzymes and enhanced hepatic antioxidant and free radical scavenging activities [26, 27], which was consistent with our observations. In the current study, our data showed that the activities of SOD, GSH, and GSH-Px were significantly decreased in the injured livers, indicating that the liver redox state was damaged. However, the levels of SOD, GSH, and GSH-Px were increased in the mice pretreated with SLE in a dose-dependent manner after CCl_4_ injection. These results demonstrate that SLE effectively attenuated CCl_4_-induced oxidative stress in the mice via enhancing the antioxidant capability. Furthermore, several reports showed that SLE could inhibit oxidative stress in CCl_4_-induced liver injury by inhibiting CYP450 enzymes [28, 29]. Since CYP2E1 is the major isozyme involved in the metabolism of CCl_4_, the expression of CYP2E1 was investigated. A similar result was obtained in this study. Expression of CYP2E1 protein was decreased after CCl_4_ treatment, but SLE blocked this decline in CYP2E1 expression. In addition,* S. chinensis* may activate the Nrf2-ARE pathway to induce antioxidant effects to prevent liver toxicity induced by acetaminophen (APAP) [30]. These studies suggested that SLE could suppress oxidative stress induced by hepatotoxic chemicals.

CCl_4_-induced liver injury was reported to be associated with inflammatory response [31, 32]. CCl_4_ and excessive free radicals probably activate kupffer cells, which can mediate the hepatic inflammation process by producing proinflammatory cytokines, such as TNF-a, IL-1*β*, and IL-6. TNF-a is a main proinflammatory cytokine and plays an important role in cell death, immune cell activation, and inflammation through different signaling pathways. In our study, the mRNA level and serum content of TNF-a were significantly increased after CCl_4_ treatment, which was attenuated by SLE treatment in a dose-dependent manner. Our results also demonstrated that the mRNA levels of other inflammatory cytokines, IL-1*β* and IL-6, were remarkably decreased by SLE treatment in mice with CCl_4_-induced liver injury. To further study the anti-inflammatory effect of SLE, NF-*κ*B signaling was investigated. NF-*κ*B is famous for a transcription factor that regulates the expression of various proinflammatory cytokines in kupffer cells. Also, NF-*κ*B signaling pathway is considered a major role in the pathophysiology of liver injury. In CCl_4_-induced liver injury, NF-*κ*B was activated by CCl_4_-induced oxidative stress and inflammatory cytokines. Previous studies have found that schisandrin exerts its anti-inflammatory effect by modulating NF-*κ*B signaling pathway in vitro [33–35]. In the current study, CCl_4_ treatment activated NF-*κ*B and increased p-NF-*κ*B expression, whereas SLE treatment significantly decreased the expression level of p-NF-*κ*B. Taken together, our findings demonstrate that SLE could attenuate the release of proinflammatory cytokines and inhibit the activation of NF-*κ*B in the CCl_4_-induced liver injury.

Additional studies have focused on elucidating how SLE protects against hepatocyte death. Apoptosis and necrosis are two patterns of cell death. It is widely believed that necrosis is the main way of cell death in CCl_4_-induced hepatocyte death. However, several studies have reported that severe apoptosis of hepatocytes was involved in CCl_4_-induced acute liver injury [8, 36, 37]. Reactive oxygen species produced by oxidative stress can induce intrinsic mitochondrial apoptosis and ultimately lead to caspase-dependent apoptotic signaling [38]. The Bcl-2 family is the dominant regulators in the process of mitochondrial apoptosis. In this study, we found that CCl_4_ increased the number of TUNEL-positive cells compared with the normal mice, but SLE pretreatment decreased this trend. Furthermore, the expression levels of Bcl-2, Bax, and cleaved caspase-3 were investigated by Western blot. As expect, CCl_4_ treatment increased the Bax expression and downregulated the Bcl-2 expression, which could be reversed by SLE. Meanwhile, the cleaved caspase-3 expression was decreased by SLE. These results support that SLE protects the liver against hepatocyte apoptosis during CCl_4_-induced liver injury.

To further investigate the effect of SLE on hepatocyte apoptosis, we examined the JNK signaling pathways, knowing that JNK signaling plays a critical role in CCl_4_-induced mitochondrial dysfunction and hepatocyte death [15]. In this study, we found that the phosphorylation level of JNK was increased by CCl_4_ compared with the control group. However, SLE treatment significantly decreased the phosphorylation level of JNK. How does SLE suppress JNK signaling in the CCl_4_-treated mice? These results showed that SLE suppressed JNK signaling pathway by suppressing the phosphorylation of JNK. In addition, it has been demonstrated that oxidative stress can activate JNK to cause apoptosis by mitochondrial intrinsic apoptotic pathways [12, 39]. Sustained JNK activation contributes to Bcl-2 family-mediated apoptosis and mitochondrial permeability transition (MPT) induced necrosis [40]. In this study, CCl_4_ treatment increased oxidative stress, thereby activating JNK and downstream signaling pathways. Correspondingly, SLE suppressed JNK activation in the CCl_4_-treated mice, which may be associated with decreased oxidative stress. Taken together, our data indicated that SLE ameliorates hepatocyte apoptosis maybe through regulating JNK signaling pathway.

## Figures and Tables

**Table 1:** Primer sequences for real-time PCR assay.

Gene (ID)	Primer sequences (5′-3′)	Product length (bp)
TNF-a (21926)	Forward: GACGTGGAACTGGCAGAAGAG Reverse: TTGGTGGTTTGTGAGTGTGAG	228
IL-1*β* (16176)	Forward: GAAATGCCACCTTTTGACAGTG Reverse: TGGATGCTCTCATCAGGACAG	116
IL-6 (16193)	Forward: CCAAGAGGTGAGTGCTTCCC Reverse: CTGTTGTTCAGACTCTCTCCCT	118
GAPDH (14433)	Forward: AGGTCGGTGTGAACGGATTTG Reverse: TGTAGACCATGTAGTTGAGGTCA	123
